# Addressing Trauma-Informed Principles in Public Health through Training and Practice

**DOI:** 10.3390/ijerph19148437

**Published:** 2022-07-11

**Authors:** Shan Parker, Vicki Johnson-Lawrence

**Affiliations:** 1 Department of Public Health and Health Sciences, University of Michigan-Flint, Flint, MI 48502, USA; 2 Department of Family Medicine, Michigan State University, East Lansing, MI 48824, USA; john4093@msu.edu

**Keywords:** trauma-informed, collective trauma, public health training, Flint Water Crisis

## Abstract

The increasing prevalence of traumatic events requires our public health workforce to be knowledgeable about ways trauma influences population and individual health. There is a gap in student training about the various ways that traumatic events affect their capacity to perform public health work and the communities they serve. While other human services disciplines explicitly use trauma-informed terminology and concepts in student training, references to trauma-informed approaches are more implicit in public health curricula. This study examined trauma-informed principles and related terminology for use in public health coursework in the context of a community-wide water contamination public health crisis in Flint, Michigan, USA. We addressed the principles of trauma-informed approaches across key competency areas common to USA public health accredited programs, including discussion to support student understanding of the principle in action. Using trauma-informed language (1) enhances our capacity to name and respond empathetically in traumatized communities, (2) provides guiding principles for less community-engaged efforts, and (3) fosters stronger relationships for more community-engaged initiatives by providing areas of accountability for unintended consequences throughout the program’s development and implementation processes. Rising public health professionals equipped with knowledge of trauma-informed approaches can more intentionally minimize unintended negative consequences of public health initiatives.

## 1. Introduction

Public health students need preparation to perform public health work without causing harm in communities affected by high rates of trauma. Public health practice includes the delivery of health interventions in diverse communities, and university-based public health programs lead the training process. The COVID-19 pandemic has been a global reminder of the collaborative and cross-disciplinary efforts required for effective public health practice through traumatic events. COVID-19 has negatively affected morbidity and mortality rates [1], mental health [2], and social experiences including social isolation [3]. COVID-19 was superimposed on existing complex, traumatic, community-wide chronic stressors including racism [4,5,6], poverty [7], gun violence [8], family substance abuse [9], and other historical collective traumas [10] that have been acknowledged as stressors, social determinants, and public health issues. Experiencing a series of traumatic events has negative immediate and long-term effects on mental health [11] and physical health [12]. Collective traumatic experiences have and will continue to shape the public health environment, and public health students need preparation to serve in affected communities.

According to the US-based Association of Schools and Programs of Public Health (ASPPH) academic program finder index of the 2580 CEPH accredited programs, 938 (36%) included “community trauma” as a search keyword, 872 (34%) included “traumatized populations” as a keyword, just 148 (6%) programs included “collective trauma” as a keyword, and 53 (2%) included “traumatic stress” as a keyword [13]. These search data reflect some level of awareness of community trauma across public health education programs but may also reflect a continuing need to address traumatic stress and collective trauma in public health work, including impacts on the communities, workforce capacity, service provision, and how students can prepare themselves to act with empathy and compassion—personally and professionally—when performing public health work in communities affected by multiple complex traumas.

The Substance Abuse and Mental Health Services Administration (SAMHSA), the leading federal agency addressing mental health services in the USA, define trauma as “an event, series of events, or set of circumstances that is experienced by an individual as physically or emotionally harmful or threatening and that has lasting adverse effects on the individual’s functioning and physical, social, emotional, or spiritual well-being” [14] (p. 7). Trauma is often framed as an exposure that needs to be mitigated using public health prevention strategies. The typical public health coursework discussion of trauma addresses the bodily system (psychological or physiological) affected or the intensity (acute/singular/ongoing) of the traumatic event. Collective traumas are commonly framed as widespread stressors or social determinants of population health that reflect inequitable power dynamics and resulting decisions that negatively affect some marginalized population. Traumatic experiences that are directly tied to institutional decisions/policies can result in negative perceptions and distrust of the affiliated decision-makers; they can also contribute to health inequities. We have to maintain awareness that collective traumas are still individual-level experiences that have a long-term influence on personal health beliefs, attitudes, and actions.

As public health faculty in Flint, MI, USA, we have been challenged to deliver training experiences that prepare our students to meet public health needs in Flint. The Flint community has navigated multiple collective traumas that still affect the community, including long-term economic instability [15], the more recent 2014–2016 Flint Water Crisis (FWC), in which city residents were exposed to high lead levels through water contamination [16], and then, COVID-19 in 2020. During the FWC, our faculty integrated content about the impacts of collective stressors on public health practice within their sub-disciplines. Learning from our own community engaged work, we observed that even well-meaning evidence-driven public health actions were met with mixed response, at least partly attributable to high institutional distrust because of the FWC.

Learning from our professional colleagues in human services and clinical care settings, we adopted SAMHSA’s concept of trauma-informed approaches [13] and guiding principles of trauma-informed practice [14].

### 1.1. Trauma-Informed Approaches

Trauma-informed approaches (TIA) are evidence-driven practices to support the health and wellbeing of patients and clients managing traumatic experiences by avoiding re-traumatization. “A program, organization, or system that is trauma-informed realizes the widespread impact of trauma and understands potential paths for healing; recognizes the signs and symptoms of trauma in staff, clients, and others involved with the system; and responds by fully integrating knowledge about trauma into policies, procedures, practices, and settings” [13] (p. 9). Following these principles and guidelines, the concept of TIA aligns closely with public health frameworks addressing socioecological and multilevel determinants of health that are frequently employed to develop public health programming in vulnerable and distressed communities.

### 1.2. Trauma-Informed Principles

SAMHSA provides six principles to inform TIA development: (1) safety—minimizing risk and building one’s sense of control; (2) trustworthiness and transparency—ensuring strategies and decisions are visible, described clearly, and do not violate trust in relationships; (3) peer support—voluntarily building mutual and respectful relationships; (4) collaboration and mutuality—rebalancing power differentials in decision-making; (5) empowerment, voice, and choice—acknowledging strengths and having space to use them; and (6) recognizing cultural, historical, and gender issues—avoiding stereotypes, promoting nurturing cultural practices, and addressing historical trauma [14] (p. 11). Trauma-informed perspectives are becoming more common in public health work [17,18,19], and trauma-informed principles already overlap with tenets of public health anchored in participatory methods.

Several human services and public-health adjacent disciplines have adopted trauma-informed principles to offer better (and cost-saving) services to their clients, including health care [20], social work [21] and justice settings [22], and education [23]. These professionals have adopted the language of trauma-informed care to reflect their efforts to provide services, often in traumatized populations, without re-traumatizing individuals in the process. While other disciplines explicitly use trauma-informed terminology and concepts in their training, references to trauma-informed approaches have been more implicit in public health curricula, but the terminology use has expanded. Of the 2560 CEPH accredited public health programs, 73% used the keyword “trauma-informed” for their program in the ASPPH index [13]. We used these principles to guide our public health practice to better ensure that our wide-reaching public health work was not creating harmful unintended consequences under the guise of public benefit. As the prevalence of collective traumatic events continues to rise, we recognize the importance of providing students with these trauma-informed principles to reduce the likelihood of re-traumatization through their public health work.

This manuscript explores how trauma-informed principles and terminology can be incorporated into public health student coursework using FWC events as examples. We discuss how trauma-informed principles align with community-engaged public health practices to drive equitable and inclusive processes and health outcomes. This work is to ensure that the lessons we learned through Flint-based work for minimizing unintended consequences are accessible to public health educators seeking strategies to reduce harm in public health.

## 2. Materials and Methods

We first provide an overview of the connections between trauma-informed principles and existing public health training content related to community engagement and equity/inclusion with regard to both engagement processes and health outcomes (Table 1). The second step was to address the principles of trauma-informed approaches across key competency areas common to USA public health accredited programs, including discussion to support student understanding of the principle in action in Table 2, Table 3, Table 4, Table 5, Table 6 and Table 7.

### 2.1. Community Engagement Approaches

Our experiences working in the Flint, MI community were vividly shaped by historical and pressing community-wide experiences that were traumatic for many residents. Informed by the history of community health inequities, we adopted community-engaged approaches to partner with local residents toward developing beneficial, effective, and sustainable public health programs. The engagement continuum [24,25] can be as minimal as seeking out community expertise concerning program ideas, and as involved (participatory) as employing community members as decision-making partners that also develop, implement, and evaluate programming. Community engagement levels vary widely but regularly include participatory approaches for information gathering, equitable and inclusive problem solving and solution-building processes, and expectations of equitable improvements in public health outcomes.

Numerous decisions of the past have been made with limited attention to the public health impact across diverse communities and, subsequently, require significant investment to correct. To reduce unintended, avoidable, and negative consequences, decision-making should include an array of stakeholders from the communities that are impacted by the decisions being made. In practice, this means engaging potential users in the development process of public health programs/interventions to avoid barriers to program use. Engagement and participatory practices have been recognized as critical to improving health and health equity [26], including by funding agencies (Patient-Centered Outcomes Research Institute) and foundations (e.g., Robert Wood Johnson Foundation).

### 2.2. Equitable and Inclusive Processes

These processes are necessary in nearly all forms of public health practice. Every organization and program uses processes for decision-making in the course of public health work. Such processes commonly include defining public health goals and needs, conceptualizing service offerings, selecting implementation strategies, and evaluation. Even without malicious intent, decision makers rely on information they deem relevant. Using community engagement approaches, decision makers can build (their) awareness about the potential effects (unintended consequences) of their program decisions. Engaged stakeholders can raise their concerns, inform solutions, or be partners for program implementation. While some processes can yield similar outcomes across different communities, other processes may need modification to achieve the desired health outcomes. Importantly, the final decisions should reflect attention to the needs of the communities being served. In vulnerable communities, additional consideration is necessary to ensure the request for engagement does not generate greater vulnerability. Given process work requires ongoing engagement and proper compensation for community members’ time, and expertise is warranted.

### 2.3. Equitable and Inclusive Outcomes

There is a broad range of primary, secondary, and tertiary outcomes associated with public health practice. Furthermore, communities can have notably different expectations about what they determine as the benefits and important outcomes of public health programs and interventions. For example, communities with high disease rates may benefit from medication adherence interventions, but they may also benefit from transportation improvement (to see a health provider) or eliminating disease-related community exposures. Engaged community stakeholders can support identification of key impactful outcomes based on their experiences. They can also highlight process outcomes that may need to be addressed in order to reach the public health goal, and likely have greater community participation along the way.

### 2.4. Connecting Public Health Approaches with Trauma-Informed Principles

Table 1 reflects connections and mutual purposes between trauma-informed principles and existing public health approaches centered in community engagement, equitable and inclusive processes, and equitable and inclusive health outcomes. Our use of community engaged approaches aligns with principles of (1) trustworthiness and transparency, (2) collaboration and mutuality, and (3) empowerment, all focused on acknowledging community expertise, using clear language, and ensuring key concerns are not overlooked. Our attention to equitable and inclusive processes and planning aligns with the trauma-informed principles of safety and peer support, reflecting shared intent to ensure decision-making is deliberate to yield beneficial impact. The connection between equitable and inclusive health outcomes and recognizing cultural, historical, and gender issues reflects shared attention to the range of metrics that can be used to measure health outcomes and the appropriateness of existing metrics across community subpopulations.

### 2.5. Incorporating Trauma-Informed Principles into Public Health Curricula

Accredited public health training programs use competency-based models to ensure students can demonstrate their capacity as practitioners. The Council on Education for Public Health agency is the nationally recognized accrediting body for schools of public health and for public health programs outside schools of public health in the USA [27]. Undergraduate public health program concepts address advocacy, community dynamics, critical thinking, cultural context, ethical decision making, work ethic, networking, organizational dynamics, professionalism, research methods, systems thinking, and teamwork/leadership. The MPH foundational competencies include evidence-based approaches to public health, public health and health care systems, planning and management to promote health, policy in public health, leadership, communication, interprofessional/intersectoral practice, and systems thinking. The criteria reference social and behavioral factors in relation to population health, but do not explicitly address response strategies as a competency.

These competencies provide spaces within public health curricula to incorporate trauma-informed principles, terminology, and practice. As students develop competencies to perform public health work, it is critically important that they also recognize potentially harmful and unintended consequences of their decisions and actions on vulnerable and traumatized communities. Trauma has been addressed across core knowledge areas of public health, including biostatistics [28,29], epidemiology [30], social [31] and behavioral [32,33] sciences, health services administration [34,35], and environmental health sciences [36]. The discussion of trauma-related health outcomes can vary across disciplines. For example, work in epidemiology and biostatistics may focus on exposure measurement and statistical modeling, health services work may focus on treatment quality, and environmental health work may investigate the source of exposure.

In nearly any case, the goal of public health practices is to improve public health conditions. No public health disciplines are removed from community engagement processes and information gathering. In vulnerable communities, resident expectations may include inclusive problem solving and solution-building practices to achieve, not just better, but equitable improvements in health outcomes.

Trauma is an unavoidable component of public health work, especially in addressing health disparities, inequities, and social determinants of health. Having and using a framework of trauma-informed principles provides lasting guidelines for community engaged work that is less likely to cause unintentionally harder circumstances for people affected by traumatic events.

### 2.6. The Flint Water Crisis as a Learning Example

This background information is our reference example for applying trauma-informed principles with public health competency areas. The FWC grabbed national attention in 2015 as evidence emerged of lead-poisoning resulting from a 2014 water source shift [16]. In 2014, Flint residents and community activists presented at city council meetings with yellowish-brown tap water. State-led water quality tests showed no actionable contaminant levels. Independent testing led by a citizen–academic scientist partnership [37] in August 2015 showed actionable contaminant levels (greater than 15 parts per billion) based on the Michigan Lead and Copper Rule, and clinical research by a Flint-based pediatrician [38] showed high lead levels in area pediatric patients.

Negative psychological effects [32,39] emerged alongside the quickly recognized negative physiological effects [38] resulting from the FWC. Negative social effects, and particularly community distrust of local public institutions [40], soared and reminded many of the historical institutional disinvestment in the Flint community. The trauma associated with the FWC was direct and vicarious: community advocates were ignored from 2014–2015 when they presented water quality concerns to elected leaders [41] and families were anxious about lead exposure and related health damage for themselves and their loved ones. Community providers across clinical and non-clinical disciplines became responsible for mental health promotion of their service populations.

Area activists and scientists pushed for government action to improve municipal water quality; the quality was improved to meet state and federal standards by July 2016 [42]. Local workgroups collaborated with state officials to revise the Michigan Safe Drinking Water Act in 2018 [43]. The revision modified the Lead and Copper Rule to lower the action level for lead in drinking water from 15 to 12 parts per billion, effective 2025.

## 3. Results

### 3.1. Trauma-Informed Principles for Community Engagement in Public Health

Engaging community members as stakeholders in program management where their expertise is respected represents *collaboration and mutuality* when power differentials are leveled. When stakeholder input is meaningfully incorporated, community members may feel more *empowered to voice* their perspectives. Ongoing engagement that addresses intent and motivation builds *trustworthiness and promotes transparency* for external stakeholders, which is particularly important in communities with high levels of institutional distrust. Maintaining space for community engagement in public health practice fosters *safety and a sense of control* about the program components, and over the longer term, encourages *peer support and voluntary relationship* building with stakeholders from various backgrounds. Tailoring the program approaches across diverse community settings can be a form of *recognition of issues related to culture, gender, and historical trauma*. When engagement is an iterative process, decision makers can grow to be more mindful about the community impact of decisions being made when external stakeholders and community members are not engaged. With empowered stakeholders at the table, we can ensure the evaluation and data interpretation processes are transparent and properly framed and represent inclusive and realistic outcomes across community subgroups.

### 3.2. Trauma-Informed Principles in Public Health with Examples from the Flint Water Crisis

With the FWC as an example, Table 2, Table 3, Table 4, Table 5, Table 6 and Table 7 demonstrate how trauma-informed principles can align with public health foundational competency areas [40] and how those principles can be integrated into public health course work. In the training setting, the instructor can work through examples in each competency area to address traditional public health questions (In Class Reflection Questions) with an iterative approach. As we generate initial answers to the questions, we can determine whether any of the trauma-informed principles are violated (Public Health—Trauma-Informed Principles to be Integrated into Course) and revise the answers to lessen potential harm. We can then use equitable and inclusive thinking to evaluate the improved answers—do we really understand how the service recipients will experience our recommendation? Are there groups that will have poorer experiences because of our recommendation, and will the health outcomes be similarly improved across community subgroups? In these discussions (“Reflections from the FWC”), the instructor has the opportunity to use the language of the trauma-informed principles to emphasize intentionality to avoid re-traumatization, then use the same trauma-informed thinking to revise the recommendations, especially in communities managing trauma (Summary Points for Student Learning). Across all of these examples, it will be necessary for the instructor to tailor the depth of the discussion based on relevance and course level.

### 3.3. Other Strategies for Integrating Trauma-Informed Principles into Public Health Curricula and Practice: Development of Community Engaged Public Health Courses

While public health students attending any institution need to have a strong grasp on course content, students at institutions in vulnerable communities such as Flint must also understand the strengths and challenges of the social landscape where the institution is located when they live, work, and play there. Courses designed to address current events are opportunities to connect our public health competencies with trauma-informed principles, particularly if the participating students are entering the workforce during an ongoing traumatic event (such as the COVID-19 pandemic or FWC). At the University of Michigan—Flint, a free community-centered course with a panel format and small group discussions was developed to facilitate bidirectional learning between policy makers, public health practitioners, community members, and students. With this approach, we advanced the reach of traditional public health course materials outside of the classroom and into community spaces to intentionally support participatory engagement, equitable, and inclusive information processing and discussing outcomes important to diverse communities impacted by the FWC. This is trauma-informed thinking in public health practice. There are certainly opportunities to integrate trauma-informed thinking into public health curricula in other forms, and these will be examined in future studies.

## 4. Discussion

The increasing prevalence of traumatic events requires our public health workforce to be knowledgeable about ways trauma influences health at population and individual levels. Collective traumatic events have an impact across public health sub-disciplines, and students who are equipped with knowledge of trauma-informed principles may be better able to navigate complex public health actions. The main implication of this work is that the language of trauma-informed approaches belongs in public health content and aligns with existing public health competencies. Trauma-informed content is becoming more common in professional degree programs, including in medical education [44] and social work [45]. Cross-disciplinary professional development resources already exist [46,47,48,49,50,51] for using trauma-informed strategies in various settings. Similar content can be developed and tailored for use among public health professionals. This can be especially helpful when working in traumatized community settings/environments affected by traumatic stressors originating from systemic policies (e.g., impoverished and racial minority communities).

The second implication references the benefits of using the language and terminology of trauma-informed approaches. Using trauma-informed language to characterize major events as traumatic stressors signals public health workers to recognize the potential for major impact in their professional capacities and to take action, including decision-making [52], with empathy. In their work, however, being trauma-informed requires intentional effort to not bring unintended harm to communities where we serve. Public health professionals can use principles of trauma-informed care to guide their approach to inclusive processes for information gathering, problem solving, solution-building, and decision-making while minimizing unintended negative consequences. In resource-constrained environments, using shared language allows professionals across disciplines to maximize collaborative impact by avoiding duplication of services based on discipline-specific terminology. Trauma-informed practices will reflect sensitivity to residents to take new actions, attend meetings, adopt unsustainable lifestyle changes, and address decision fatigue, even in the most well-meaning efforts.

The third implication of using trauma-informed language is centered on community-engaged efforts. Using trauma-informed language also reflects an intentionality in the process of community partnering as in existing public health work [53]. For initiatives with low community engagement, the principles of trauma-informed approaches can be used to identify and limit unintended consequences related to each principle. It also fosters stronger relationships for more community-engaged initiatives by providing areas of accountability for unintended consequences throughout the program’s development and implementation processes. This demonstrates thoughtfulness and intentionality to community partners, particularly when these principles are articulated in working agreements between community partners and institutional staff.

Future work can address training around trauma-informed practice in preparations for experiential learning experiences such as internships, capstones, culminating experiences, service learning, and even volunteerism settings where complexity and trauma are common, as in social work [54]. Rising professionals can use trauma-informed terminology and thinking to empathetically communicate with others, including distressed co-workers and clients, during stressful situations. They can utilize trauma-informed principles to guide their decisions as they engage in communities.

From our work through the FWC, and as evidenced by workforce wellness concerns during COVID-19 [55], the need to manage personal wellbeing while working in crisis conditions cannot be overlooked in professional development. Shifts in public health work in response to a traumatic event also signify the need for precaution around self-care and sensitivity to burnout, as has happened with COVID-19 [56]. In addition, trauma-informed communication can foster better workplace relationships in traumatized community settings [57]. Finally, for non-clinical public health service providers, the content should address self-care, establishing boundaries, managing work, and workload expectations, recognizing how personal stress or grief experiences influence their professional capacity. The content should also address the causes and signals of distress in helping professions such as social work and clinical care, including moral injury, burnout, and secondary trauma [58,59,60,61].

Future research is warranted to evaluate the practice and implications of using trauma-informed approaches in public health. This includes evaluation of our disciplinary integration of trauma-informed approaches, language, and community engagement practices into the student training process that is currently guided by competency-based education. This research should also address public health use of trauma-informed approaches with adjacent human services disciplines, including the use of appropriate metrics for health, process, community, and population outcomes.

## 5. Conclusions

Rising public health professionals will be leading solutions to respond to widespread collective experiences that negatively impact population health. They will be expected to understand how and why diverse communities deserve tailored, inclusive, and trauma-informed programs. This preparation can begin or be enhanced by intentional introduction of trauma-informed terminology, principles, and situational examples throughout the public health curriculum. This provides anchoring concepts to inform their thinking about the approach to serving individual clients and communities. It exposes them to the many existing resources, such as those from SAMHSA, designed to support people working in helping professions to provide effective services and support their professional capacity.

Other service disciplines have integrated trauma-informed approaches into their curricula and public health could benefit from the frameworks that have been developed for use in those disciplines. It is imperative that we are educating future practitioners to address public health issues from a trauma-informed lens to improve community engagement and health outcomes while not adding to or re-traumatizing the communities in which they serve.

## Figures and Tables

**Table 1:** Connecting Public Health Approaches with Trauma-Informed Principles.

Public Health Approach	Trauma-Informed Principles	Mutual Intent
Community Engagement	Trustworthiness and transparency (ensure understanding with respect) Collaboration and mutuality (rebalance power differentials) Empowerment, voice, and choice (strengths-focused)	Acknowledging community experiences as expertise Use clear and shared language Recognize diversity in expertise of impacted individuals Raise concerns that would otherwise be overlooked
Equitable and Inclusive Processes	Safety (sense of control) Peer support (relationship)	Ensure decisions yield personal and population level improvements Incorporate contextual experience as formal information in decision-making Use shared knowledge to improve sustainable decision-making
Equitable and Inclusive Health Outcomes	Recognizing cultural, historical, and gender issues (avoid stereotypes)	Examine multiple definitions and metrics of improvement

**Table 2:** Trauma-Informed Principles in Public Health: Addressing Public Health Leadership with Examples from the Flint Water Crisis.

Competency Area: Leadership	The focus is to create a vision, empower others, foster collaboration, and guide decision making utilizing negotiation and mediation skills to address organizational and community challenges
In Class Reflection Questions	Did decision-makers engage (or dismiss) groups of people experiencing a common health concern? Did they work together to find the problem and identify solutions?
Public Health-Trauma-Informed Principles to Integrate	Community Engagement Trust and Transparency Collaboration and Mutuality
Classroom Translation and Discussion	
Reflections from the FWC	While citizens and activists presented local officials with clear evidence of discolored water, a citizen–academic scientist partnership was established to facilitate independent data collection about lead levels Discussions were needed where all parties were heard, experiences were validated, and respect for community expertise and their lived experiences was demonstrated
Summary Points for Student Learning	Leaders must ensure we have participatory engagement, transparency, collaboration, and mutuality to respond to public health concerns. These features are necessary to clearly describe the public health problem, potential actions, and identify potential consequences related to next steps

**Table 3:** Trauma-Informed Principles in Public Health: Addressing Public Health Planning and Management with Examples from the Flint Water Crisis.

Competency Area: Planning and Management	This area focuses on community assessment of health while applying awareness of cultural values and practices in the design or implementation of public health policies or programs and evaluation of programs. The area addresses the design of population-based policies, programs, or interventions. Also addressed is budget and resource management
In Class Reflection Questions	Do we have preparedness plans for potential public health crises? How do we integrate lessons from previous similar events? Do we have adequate resources to mitigate negative effects of local crises?
Public Health-Trauma-Informed Principles to Integrate	Equitable and Inclusive Process Trust and Transparency Recognizing Cultural, Historical, and Gender Issues
Classroom Translation and Discussion	
Reflections from the FWC	The long history of social and economic disinvestment and high community distrust in the city of Flint influenced community expectations around public health. The crisis weakened community preparedness to mitigate damage related to the water crisis. Resources need to be distributed according to the problem severity in seeking parity when the goal is community health To achieve public health goals that are relevant, practitioners must understand and acknowledge the historical, societal, governmental, and economic trauma of the community. For example, for years, community members were inadequately compensated for their work on community problems, often without receiving payment or lesser pay for the same work that researchers, community organizations, and other officials received for doing the same work
Summary Points for Student Learning	Vulnerable communities with a history of inequitable health outcomes will need additional resources to address existing and arising problems. Health improvement goals must account for existing community health status. This work should also address historical and external initiatives that were framed to be helpful but contributed to community disenfranchisement from broken promises

**Table 4:** Trauma-Informed Principles in Public Health: Addressing Evidence-Based Approaches and Policies in Public Health with Examples from the Flint Water Crisis.

Competency Area: Evidence-Based Approaches and Policies	This area focuses on application of epidemiological methods and the collection, analysis, and interpretation of both quantitative and qualitative data collection methods for public health research, policy, or practice.
In Class Reflection Questions	What are the different implications of the data we have available? Why will residents volunteer their personal health data? How are my beliefs contributing to my interpretation of the data? Have we considered context in the data analysis and interpretation?
Public Health-Trauma-Informed Principles to Integrate	Equitable and Inclusive Process Safety
Classroom Translation and Discussion	
Reflections from the FWC	The assessment protocol for identifying excess lead and contaminant exposure did not address the expectations of those most impacted. Residents consumed water from their household tap, but contaminant assessment happened at the water source. The assessment process required modification because it did not consistently reflect the water quality as experienced by the residents. Household water infrastructure was damaged by water contaminants and lowered water quality after treatment protocols were put in place. Residents needed clear rationale about allowable risks with exposure to dangerous conditions.
Summary Points for Student Learning	One data source or data collection method can generate different implications for policy makers and community stakeholders. Personal perceptions of the relevance and value of different data can bias the interpretation of results. Defining safety, particularly about exposures, can vary significantly for policy makers and the consumers who experience related health consequences.

**Table 5:** Trauma-Informed Principles in Public Health: Addressing Public Health Policy with Examples from the Flint Water Crisis.

Competency Area: Policy	The focus is to address the policy making process, coalition, and partnership building to influence public health outcomes. Ethics, advocacy for political, social, or economic policies and programs that will improve health in diverse populations and the evaluation of policies for their impact on public health and health equity are addressed.
In Class Reflection Questions	Are policies being used to restrict accessibility to solutions across different audiences? Are policies going to be enforced to yield similar outcomes across audiences? How will the policies be enforced?
Public Health-Trauma-Informed Principles to Integrate	Equitable and Inclusive Outcomes Empowerment, Voice and Choice
Classroom Translation and Discussion	
Reflections from the FWC	Local workgroups collaborated with state officials to revise the Michigan Safe Drinking Water Act in 2018. The revision modified the Lead and Copper Rule that lowers the action level for lead in drinking water from 15 to 12 parts per billion, effective 2025.
Summary Points For Student Learning	Several partners, including people from affected groups, should be involved in deciding the ideal outcomes to work toward. Public health leaders share in the responsibility to maintain spaces for advocacy—with and on behalf of—community members. When there are multiple ideal outcomes, the collaborators can develop—and commit to—plans that work toward the set of ideal outcomes.

**Table 6:** Trauma-Informed Principles in Public Health: Addressing Public Health and Health Care Systems with Examples from the Flint Water Crisis.

Competency Area: Public Health and Health Care Systems	Addresses the organization, structure, and function of health care, public health, and regulatory systems across national and international settings Addressed here are the means by which structural bias, social inequities, and racism undermine health and create challenges to achieving health equity at organizational, community, and societal levels.
In Class Reflection Questions	What will health care systems require to address this public health issue? Are these efforts centered on primary, secondary, or tertiary prevention?
Public Health-Trauma-Informed Principles to Integrate	Equitable and Inclusive Process Collaboration and Mutuality
Classroom Translation and Discussion	
Reflections from the FWC	Clinical research identified the public health problem with objective data. Primary prevention initiatives were implemented in community and clinic settings to promote health and positive health behaviors. Additional clinical screening services were warranted to identify and treat adults and children affected by lead exposure. Mental health services were necessary to address psychological stress for parents and children.
Summary Points for Student Learning	Decision making processes can benefit from cross-disciplinary perspectives to identify necessary resources to achieve the intended outcomes. Decision making should include the people, institutions, and agencies that will be expected to act. We need to acknowledge and plan for resource limitations and the related consequences. Disjointed multi-system efforts will be the most confusing for community members. Some services will be duplicated to ensure reach into different sub-communities.

**Table 7:** Trauma-Informed Principles in Public Health: Addressing Public Health Communication with Examples from the Flint Water Crisis.

Competency Area: Communication	The focus is on audience appropriate communication strategies and, specifically, the importance of cultural competence in communicating public health content. The importance is to whom we communicate the public health message, the appropriateness and validity of the messages communicated, and how those messages are disseminated.
In Class Reflection Questions	Was messaging consistent or tailored to different audiences? Are the expected action steps the same for different audiences? Is there shared understanding about the setting, problems, and potential solutions?
Public Health-Trauma-Informed Principles to Integrate	Equitable and Inclusive Process Peer Support Safety
Classroom Translation and Discussion	
Reflections from the FWC	For persons managing lead-related health problems, improving the standard may be more meaningful with their children and families; for people unaffected by lead-related health problems, the attention to the water quality standards may appear trivial without more discussion. The messaging about allowable risks helps consumers understand the next action steps. The information also needed to be tailored to different conditions and with different dissemination approaches (considering language, literacy, media exposure, etc.).
Summary Points for Student Learning	Policies and standards can have different implications across community subgroups. Communications have to clearly describe how the standards might require different subsequent action steps to reach meaningful and acceptable solutions. An inclusive set of partners is needed to decide on the best outcomes for the community. Such decisions cannot be derived solely from the preferences of any one group or audience.

## Data Availability

Not applicable.
